# Exosome‐related lncRNA score: A value‐based individual treatment strategy for predicting the response to immunotherapy in clear cell renal cell carcinoma

**DOI:** 10.1002/cam4.7308

**Published:** 2024-05-29

**Authors:** Zhan Yang, Xiaoting Zhang, Ning Zhan, Lining Lin, Jingyu Zhang, Lianjie Peng, Tao Qiu, Yaxian Luo, Chundi Liu, Chaoran Pan, Junhao Hu, Yifan Ye, Zilong Jiang, Xinyu Liu, Mouyuan Sun, Yan Zhang

**Affiliations:** ^1^ Department of Urology The First Affiliated Hospital of Wenzhou Medical University Wenzhou Zhejiang Province China; ^2^ Stomatology Hospital, School of Stomatology Zhejiang University School of Medicine, Zhejiang Provincial Clinical Research Center for Oral Diseases, Key Laboratory of Oral Biomedical Research of Zhejiang Province, Cancer Center of Zhejiang University, Engineering Research Center of Oral Biomaterials and Devices of Zhejiang Province Hangzhou Zhejiang Province China

**Keywords:** ceRNA network, clear cell renal cell carcinoma, exosome, lncRNA, machine learning, precision medicine, tumor microenvironment

## Abstract

**Background:**

Exosomes play a crucial role in intercellular communication in clear cell renal cell carcinoma (ccRCC), while the long non‐coding RNAs (lncRNAs) are implicated in tumorigenesis and progression.

**Aims:**

The purpose of this study is to construction a exosomes‐related lncRNA score and a ceRNA network to predict the response to immunotherapy and potential targeted drug in ccRCC.

**Methods:**

Data of ccRCC patients were obtained from the TCGA database. Pearson correlation analysis was used to identify eExosomes‐related lncRNAs (ERLRs) from Top10 exosomes‐related genes that have been screened. The entire cohort was randomly divided into a training cohort and a validation cohort in equal scale. LASSO regression and multivariate cox regression was used to construct the ERLRs‐based score. Differences in clinicopathological characteristics, immune microenvironment, immune checkpoints, and drug susceptibility between the high‐ and low‐risk groups were also investigated. Finally, the relevant ceRNA network was constructed by machine learning to analyze their potential targets in immunotherapy and drug use of ccRCC patients.

**Results:**

A score consisting of 4ERLRs was identified, and patients with higher ERLRs‐based score tended to have a worse prognosis than those with lower ERLRs‐based score. ROC curves and multivariate Cox regression analysis demonstrated that the score could be considered as a risk factor for prognosis in both training and validation cohorts. Moreover, patients with high scores are predisposed to experience poor overall survival, a larger prevalence of advanced stage (III‐IV), a greater tumor mutational burden, a higher infiltration of immunosuppressive cells, and a greater likelihood of responding favorably to immunotherapy. The importance of EMX2OS was determined by mechanical learning, and the ceRNA network was constructed, and EMX2OS may be a potential therapeutic target, possibly exerting its function through the EMX2OS/hsa‐miR‐31‐5p/TLN2 axis.

**Conclusions:**

Based on machine learning, a novel ERLRs‐based score was constructed for predicting the survival of ccRCC patients. The ERLRs‐based score is a promising potential independent prognostic factor that is closely correlated with the immune microenvironment and clinicopathological characteristics. Meanwhile, we screened out key lncRNAEMX2OS and identified the EMX2OS/hsa‐miR‐31‐5p/TLN2 axis, which may provide new clues for the targeted therapy of ccRCC.

## Graphical abstract

Exosome‐related lncRNAs potentially involved in ccRCC pathogenesis with distinctive prognosis and management strategy. Based on four key lncRNAs (EMX2OS, AC026401.3, AL161835.1, and AC018690.1), we constructed an exosome‐related lncRNAs score to evaluate its independent prognostic value for ccRCC patients. As the most important ERLR among the four key ERLRs, EMX2OS was screened through mechanical learning and clinical validation, and act as a ceRNA to mediate its downstream network. To further analyze immune and drug sensitivity responses to ccRCC, we finally focus on the effect of EMX2OS on hsa‐miR‐31‐5p/TLN2 axis. ERLR: exosome‐related lncRNA; GG: Golgi apparatus; MVB: multivesicular body; ICI: immune checkpoint inhibitors; ccRCC: clear cell renal cell carcinoma.

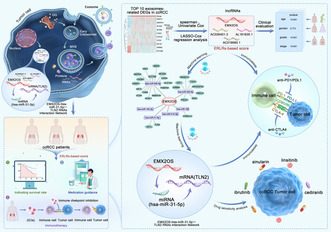

## 1 INTRODUCTION

Renal cell carcinoma (RCC) is a heterogeneous group of cancers derived from renal tubular epithelial cells. It is the seventh most common cancer in men and the ninth most common cancer in women, accounting for 3%–4% of all adult malignancies.^1^, ^2^, ^3^ Clear cell renal cell carcinoma (ccRCC), the most common pathological subtype, accounting for approximately 75% of RCCs, is characterized by a high propensity for metastasis and immune infiltration and is associated with a poorer prognosis compared to other RCC subtypes.^4^, ^5^, ^6^, ^7^ Since the clinical benefit of chemoradiotherapy is inferior to that of surgical treatment in ccRCC, surgical treatment is the main option for patients. However, 30% of ccRCC patients endure recurrence and distant metastasis after surgery.^8^, ^9^ For these patients, no effective treatments are currently available. For these reasons, the 5‐year survival rate for metastatic and advanced stage ccRCC patients is only around 13%.^10^, ^11^ In recent years, the prognosis of patients with ccRCC has improved significantly with the introduction of immune checkpoint inhibitor (ICI) therapy.^12^ However, many factors still induce metastasis and recurrence of ccRCC, with the impact of delayed diagnoses and a lack of effective disease surveillance and drug efficacy prediction methods being the most notable.^13^ There is an urgent need to develop valid screening tools and identify appropriate ccRCC biomarkers to predict the prognosis of ccRCC and improve clinical diagnosis and treatment strategies.

The tumor microenvironment is a key factor in the formation of tumors and contains a variety of potential biomarkers.^14^, ^15^ Exosomes, which are actively secreted by both normal and cancer cells, are small extracellular vesicles composed of a lipid bilayer membrane structure. They contain proteins, nucleic acids, lipids, and other bioactive substances, with various functions in the tumor microenvironment.^16^, ^17^ Exosomes have gained considerable attention in ccRCC research, both as mediators of intercellular signaling and as potential sources for the discovery of novel cancer biomarkers.^18^, ^19^, ^20^ Xia et al. found that exosomes secreted by ccRCC cells induce dysfunction of TME‐infiltrating natural killer cells, by regulating the TGF‐β/SMAD pathway and evading innate immune surveillance.^21^ Xuan et al. demonstrated that exosomal miR‐549a regulates the expression of HIF1α in vascular endothelial cells to promote angiogenesis and enhance vascular permeability, supporting promote tumorigenesis and metastasis after failed tyrosine kinase inhibitor (TKI) resistance in ccRCC.^22^ He et al. found that exosomal mRNA‐based (CUL9, KMT2D, PBRM1, PREX2, and SETD2) signatures may serve as tools for the early detection of ccRCC and differential diagnoses of uncertain renal masses.^23^ Therefore, exosomes are considered important mediators in the tumor microenvironment and are involved in regulating ccRCC tumor cell invasion, angiogenesis, and immunosuppression.^24^, ^25^, ^26^ Tumor‐derived exosomes carrying various molecules could provide a promising, non‐invasive method for the diagnosis of ccRCC.

Recently, lncRNAs have been implicated toward the multilevel regulation of exosomal genes expression, including transcriptional regulation, by recruiting chromatin‐modifying complexes and post‐transcriptional regulation by interacting with miRNAs, mRNAs, or proteins.^27^, ^28^, ^29^, ^30^ The roles of exosomal lncRNAs in cancer development have gained increasing interest. LncRNAs are primarily defined as RNAs ≥200 nucleotides long and can range up to 100 kb.^31^ The differential expression levels of lncRNAs are closely related to the occurrence and development of ccRCC.^32^, ^33^ Hu et al. found that hypoxic RCC cells secrete exosomes packaged with lncHILAR, which are taken up by normoxic RCC cells, and promote normoxic RCC cell invasion and metastasis via ceRNA for the miR‐613/206/1–1‐3p/Jagged‐1/Notch/CXCR4 axis.^34^ Zhang et al. found that lncARSR acts as a signaling molecule delivered by RCC‐derived exosomes to induce macrophage polarization by activating the STAT3 signaling pathway, thus promoting the occurrence and development of ccRCC.^35^ Therefore, exosomal lncRNAs may be involved in multiple processes of ccRCC development, including cancer cell proliferation, metastasis, drug resistance, and immunomodulation.^36^ Capturing important biological information from exosomal lncRNAs may help us better understand and manage ccRCC. However, the value of exosomal lncRNAs in ccRCC has not yet been fully revealed.

In this study, we performed systematic bioinformatics analyses to construct an exosome‐related lncRNA (ERLR)‐based score of ccRCC patients. Meanwhile, we outlined the immune infiltration landscape in ccRCC and used the ERLR‐based score to explore differences in immune cell infiltration. The aim of this study was to gain a better understanding of potential molecular immunity processes during the progression of ccRCC. Moreover, we screened for the key lncRNA through machine learning and constructed relevant competing endogenous RNA (ceRNA) networks, while attempting to identify potential regulatory connections that affect the progression of ccRCC. In addition, all the above RNA was tested in normal tissue and ccRCC tissue samples, respectively. Subsequently, based on database analysis, relevant immunoassays and drug sensitivity tests were performed. Overall, our study constructed an ERLR‐based score by screening out key ERLRs, sought out find potential ceRNA regulatory axis, and systematically analyzed key therapeutic targets, providing new clues for the clinical treatment of ccRCC.

## 2 MATERIALS AND METHODS

### 2.1 Data acquisition

RNA‐seq and miRNA‐seq transcriptome data from 539 ccRCC tissues and 72 adjacent normal tissues and the corresponding clinical information were downloaded from the Cancer Genome Atlas (TCGA) (https://cancergenome.nih.gov/) database. GSO datasets (GSE22541 and GSE167573) were downloaded from the GEO (https://www.ncbi.nlm.nih.gov/) database. GSE22541 included 44 ccRCC lung metastatic tissues and 24 primary ccRCC tissues. GSE167573 contained 63 primary ccRCC tissues and 14 adjacent normal tissues. A total of 120 exosome‐related genes were obtained from a comprehensive database of exosomes (https://www.exocarta.org/).^37^

### 2.2 Identification of exosome‐related lncRNAs (ERLRs)

First, we performed a differential expression analysis to screen for exosome‐related genes that are characteristic for the ccRCC using the R package “limma” (|log FC| >0.1 and FDR <0.05). Univariate cox regression analysis was performed to identify the prognostic exosome‐related genes using R package “survival.” The intersection genes of differentially expressed genes (DEGs) and prognostic exosome‐related genes were then incorporated into the protein–protein interaction (PPI) analysis. Ten genes, with the highest number of node connections in the protein interaction network, were identified as hub exosome‐related genes in ccRCC. Moreover, hub exosome‐related gene‐to‐lncRNA correlations were generated using Spearman correlation analysis. The lncRNAs with *R* > 0.5 and FDR < 0.001 were identified as ERLRs.

### 2.3 Construction and validation of the ERLR‐based score

The entire TCGA ccRCC cohort was randomly divided into the training cohort (*n* = 265) and the validation cohort (*n* = 265) with the ratio of 1:1. The training cohort was adopted for the construction of the prognostic ERLR‐based score, while the validation cohort was used to verify accuracy. In the training cohort, a univariate Cox regression analysis was performed to identify prognostic ERLRs. Next, based on prognostic ERLRs, LASSO regression analysis was used to choose the most significant genes by R package “glmnet” in the training cohort. Finally, these genes were incorporated into the multivariate Cox analysis to determine vital ERLRs and construct a score. The riskScore of each ccRCC patient was calculated using the followed formula:

RiskScore=e^∑CoefiExpi,

“*e*” is the natural logarithm, Coef(*i*) is the coefficient, and Exp(*i*) represents the expression of ERLRs. The median of the riskScore in the training cohort was used as the cutoff value to classify all ccRCC patients into the high‐ and low‐risk groups.

A Kaplan–Meier survival curve was drawn to display the differences in prognosis between the high‐ and low‐risk groups with the log‐rank test. Next, we implemented time‐dependent receiver operating characteristic (ROC) curves at 1‐, 3‐, and 5‐year survival, using the R package “SurvivalROC” to verify the performance of the score. Moreover, stratification analyses were generated among gender (female, male), age (≤65 or >65), grade (I‐II, III‐IV), and stage (I‐II, III‐IV) to confirm that the score was consistent across these subgroups. Also, the riskScore of the different subgroups was compared using the Wilcoxon signed‐rank test to perform an internal verification.

### 2.4 Independence test and establishment of a nomogram based on riskScore and clinical characteristics

Age, gender, grade, stage, and riskScore were included in the univariate and multivariate Cox regression analyses to screen for independent prognostic factors. A nomogram was developed using the R package “regplot” to predict the 1‐, 2‐, and 3‐year overall survival (OS) of ccRCC patients. Calibration curves were plotted using the R package “RMS” and “Survival” to evaluate the predictive power of this nomogram. Additionally, the time‐dependent ROC curves for all factors were drawn, and the area under the curve (AUC) for all factors was compared with the AUC of the score and nomogram.

### 2.5 Estimation of tumor mutational burden (TMB) and immune cell infiltrating scores

Gene set enrichment analysis (ssGSEA) was used to calculate the infiltrating scores of 23 types of immune cells, immune‐related pathways, and stromal‐related pathways using the R package “GSVA.” CIBERSOFT, as another method to evaluate the infiltration scores of immune cells, was performed to calculate the infiltrating scores of 22 types of immune cells via R package “CIBERSOFT.” In addition, the “Maftools” R package was used to evaluate mutations in different risk score groups, and two TME‐related scores (Immune score and Stromal score) were calculated by the R package “ESTIMATE.”

### 2.6 Acquisition of immunotherapy related scores

The tumor immune dysfunction and exclusion (TIDE) algorithm (https://tide.dfci.harvard.edu) was applied to obtain seven immunotherapy related scores, including ips_ctla4_pos_pd1_pos score, CD8 score, CD274 score, Dysfunction score, TIDE score, merck18 score, and IFNG score.

### 2.7 Construction of lncRNA‐miRNA‐mRNA ceRNA network in ccRCC

Importance of the identified ERLRs in prognosis was analyzed through mechanical learning with R package “randomForest,” and EMX2OS was ank1 in 4ERLRs. Downstream miRNA of EMX2OS was screened based on the mircode database (https://www.mircode.org/), and the intersection of differentially expressed miRNA in the TCGA database was obtained to identify the target miRNA. Based on the miRwalk database (https://mirwalk.umm.uni‐heidelberg.de/), criterion was set with a score = 1, and downstream mRNAs of relevant miRNAs were screened in TargetScan (https://www.targetscan.org/vert_80/) and miRDB (https://mirdb.org/). This was intersected with differentially expressed mRNAs in the TCGA database to identify downstream mRNAs. Finally, correlations with the ceRNA interaction network were established. The ceRNA network was visualized by introducing edge and nodal gene information into the interaction network with Cytoscape v3.8.0. The importance of target‐miRNAs and target‐mRNAs was analyzed through mechanical learning.

### 2.8 Function and pathway enrichment analysis

To better understand the underlying function of aberrantly expressed genes, gene ontologies (GO) were collected. We used the Database for Annotation Visualization and Integrated Discovery 6.8 (DAVID) (https://david.ncifcrf.gov/) to perform the functional analysis. Then, Kyoto Encyclopedia of Genes and Genomes (KEGG) pathways were constructed using KOBAS 3.0 (https://kobas.cbi.pku.edu.cn/anno_iden.php).

### 2.9 Survival analysis of key RNAs in the ceRNA network

Based on the expression level of key RNAs, all patients were divided into low expression and high expression groups according to the median expression level. Survival curves were drawn to compare the survival differences between the two groups.

### 2.10 Drug sensitivity and immunotherapy analysis of ceRNA network

In this study, IC50 values of drugs were obtained in Genomics of Drug Sensitivity in Cancer (GDSC) using the R package “oncoPredict.” Correlations between drug IC50 values and risk scores were analyzed by Spearman's analysis to screen for potential drugs. We then compared the differences in IC50 values between the high‐ and the low‐expression groups for drugs with absolute values of correlation greater than 0.4. The results were then visualized using the R language ggplot2. Additionally, based on the expression levels of EMX2OS and TLN2, a Kaplan–Meier survival curve was drawn to display the differences of OS between the high‐ and low‐expression groups after anti‐PD‐1, anti‐PD‐L1, or anti‐CTLA‐4 treatment‐ using the log‐rank test. Next, we implemented time‐dependent ROC curves at anti‐PD‐1, anti‐PD‐L1, or anti‐CTLA‐4 treatment using R package “SurvivalROC” to verify score performance.

### 2.11 Patients and tissue specimens

CcRCC tissues and normal control tissues were obtained from ccRCC patients undergoing radical nephrectomy in the First Affiliated Hospital of Wenzhou Medical University. This study was approved by the Human Research Ethics Committee of the First Affiliated Hospital of Wenzhou Medical University (KY2021‐160).

### 2.12 Real‐time polymerase chain reaction (RT‐qPCR) analysis

To verify the reliability of lncRNAs prognostic score in ccRCC patients, TRIzol reagent (Invitrogen, USA) was used to extract total RNA from tumor tissue and normal control tissue, respectively, and cDNA samples were synthesized using superscript II first‐strand cDNA synthesis kit (TaKaRa, Japan).^38^ The expression levels of EMX2OS, hsa‐miR‐210‐3p, has‐miR‐31‐5p, has‐miR‐183‐5p, HEYL, and TLN2 were quantified by real‐time fluorescence quantitative method. GAPDH is used as internal control.

**Table:** 

	Forward:5′ to 3′	Reverse:3′ to 5′
EMX2OS	CCAGCCAACGTCGATTTCAC	CCAGGGCCACTTGGGTATTT
hsa‐miR‐210‐3p	CGCTGTGCGTGTGACAGC	AGTGCAGGGTCCGAGGTATT
has‐miR‐31‐5p	GCGAGGCAAGATGCTGGC	AGTGCAGGGTCCGAGGTATT
has‐miR‐183‐5p	CGCGTATGGCACTGGTAGAA	AGTGCAGGGTCCGAGGTATT
HEYL	GTTCGCCATGAAGCGACC	GCCCTGTTTCTCAAAGGCAG
TLN2	ATTGTTGCCAAGCACACGTC	CTTGGCTGACTGGACGAAGT
GAPDH	AATGGGCAGCCGTTAGGAAA	GCGCCCAATACGACCAAATC

### 2.13 Statistics analysis

All statistical analyses were performed in R software (version 4.1.0), GraphPad Prism 8 (GraphPad Software, United States), and PASS 27.0 software. Each chapter introduces particular software packages utilized by R studio. The data were expressed as the mean ± standard deviation (SD) of at least three independent experiments, and the differences between the two groups were compared using the Student *t*‐test. A one‐way ANOVA with Bonferroni's multiple comparisons test was used for multiple group comparisons. Spearman's correlation coefficient test was used to evaluate the rank correlations among different variables. Kaplan–Meier survival curve was used to analyze survival differences, and the Log‐rank test was used to assess the significance of the difference in survival time between two groups. Statistical *p*‐values were subjected to two tailed tests, and *p* < 0.05 was considered statistically significant.

## 3 RESULTS

### 3.1 Screening and identification of exosome‐related lncRNAs

**FIGURE 1:**
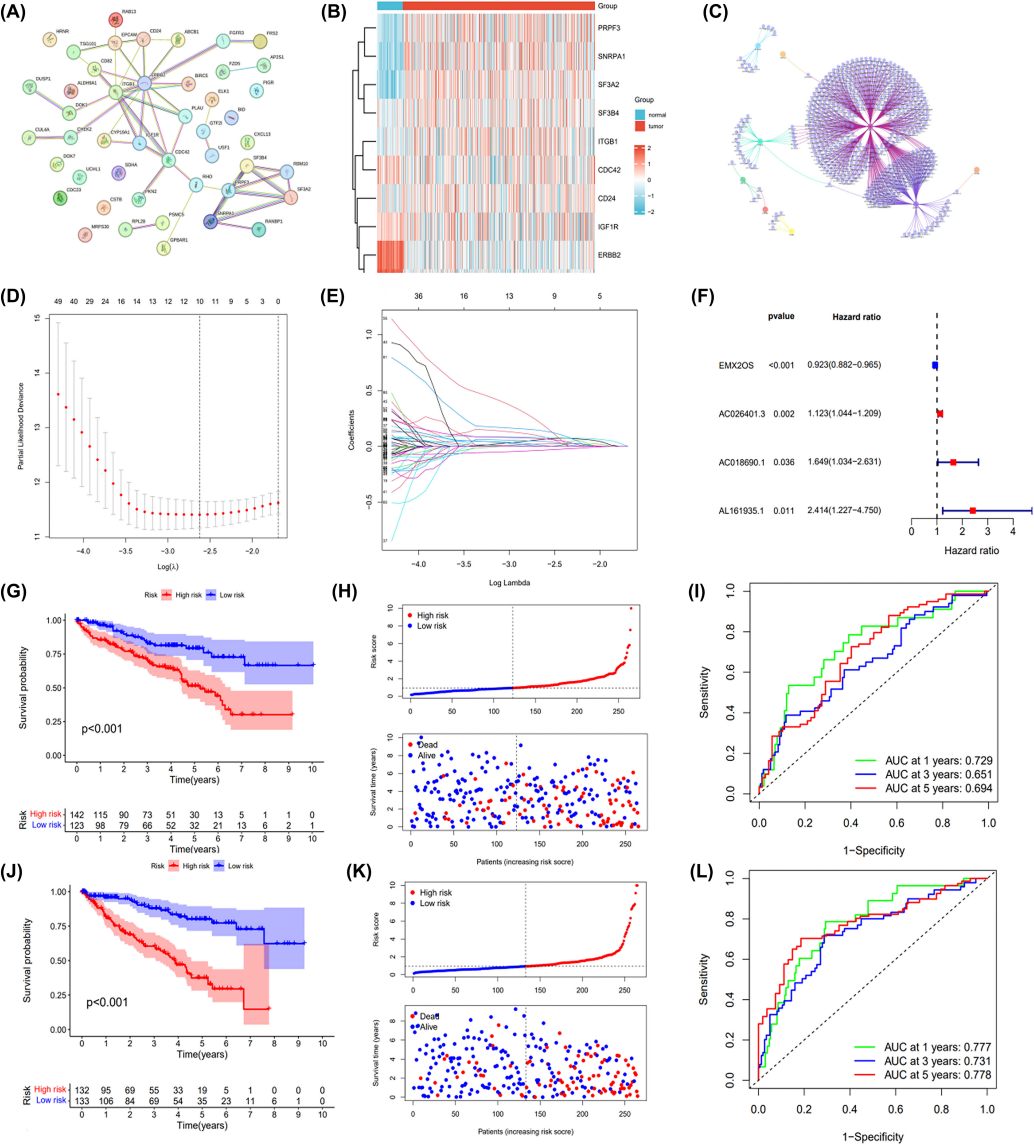
Screening and identification of exosome‐related lncRNAs. (A) PPI of 46 differentially expressed exosome‐related genes. (B) Heatmap of the TOP 10 prognosis‐related differentially expressed exosome‐related genes. (C) Co‐expression network of exosome‐related TOP 10 DEGs and exosome‐related lncRNAs. (D) Distribution of LASSO co‐efficients of exosome‐related lncRNAs. (E) The LASSO algorithm's 10‐fold cross‐validation of variable selection. (F) The multivariate Cox regression analysis of the four ERLRs. (G) Kaplan–Meier analysis of the overall survival rate of patients with ccRCC in the low‐ and high‐risk groups of the training cohort. (H) The distribution of risk score among patients with ccRCC in the training cohort. (I) ROC curve and AUCs at 1‐, 3‐, and 5‐year survival in the training cohort. (J) Kaplan–Meier analysis of the overall survival rate of patients with ccRCC in the low‐ and high‐risk groups in the validation cohort. (K) The distribution of risk score among patients with ccRCC in a validation cohort. (L) ROC curve and AUCs at 1‐, 3‐, and 5‐year survival in the validation cohort. AUC, area under the curve; DEGs, differentially expressed genes; ERLR, Exosome‐related lncRNA; LASSO, least absolute shrinkage and selection operator; PPI, protein–protein interaction network; ROC, receiver operator characteristic.

We identified DEGs in ccRCC by comparing gene expression levels in adjacent normal renal tissues using the TCGA database. A total of 99 DEGs were identified between ccRCC and normal samples (Figure S1A). Of these, 46 differentially expressed genes were associated with ccRCC exosomes and had prognostic values (Figure S1B,C). Then, we performed a protein–protein interaction network (PPI) analysis on these 46 genes and selected the TOP 10 genes for the following analysis according to differences in expression levels (Figure 1A). The differential expression of the TOP 10 exosome‐related DEGs in ccRCC was shown by heatmaps in Figure 1B. In total, 669 ERLRs were identified to be associated with top10 prognostic exosome‐related DEGs (Figure 1C). To construct the ERLRs prognostic score for forecasting the overall survival (OS) of ccRCC patients, we performed a LASSO regression and multivariate cox regression analysis with the 669 ERLRs, the four ERLRs (EMX2OS, AC026401.3, AC018690.1, and AL161935.1) were screened for the greatest prognostic value in ccRCC patients (Figure 1D,E). Univariate Cox regression analysis confirmed the prognostic value of the four ERLRs (*p* < 0.05) (Figure 1F). The whole ccRCC patients were randomly divided into two cohorts (training: validation = 1:1). Then, based on the correlation coefficient of the expression of lncRNAs in the multivariate Cox regression analysis, we construct a risk factor prediction linear model. According to the median ERLR‐based score, the patients in each cohort were divided into low‐risk group and high‐risk group (Figure 1H,K). The results of the OS Kaplan–Meier curve in the two cohorts were consistent (Figure 1G,J), and the survival rate of ccRCC patients in the low‐risk group was significantly better than that in the high‐risk group. A time‐dependent receiver operating characteristic was used to investigate the prognostic value of the characteristic (Figure 1I,L). The 1‐, 3‐, and 5‐year AUCs for the training group were 0.777, 0.731, and 0.778, respectively, while the 1‐, 3‐, and 5‐year AUCs for the validation group were 0.729, 0.651, and 0.694, respectively. These results showed that ERLR‐based score could be used as a predictor of ccRCC patients' outcomes.

To further explore the potential mechanism, we first used the R package “limma” to mine DEGs in low‐ and high‐groups, where a total of 99 DEGs were identified (Figure S2A). Subsequently, gene name and log2FC of the DEGs in the groups were used for enrichment analysis of gene ontology (GO) and KEGG pathways (Figure S2B,C). KEGG analysis also demonstrated that these genes were mainly involved in PPAR signaling pathways and metabolic pathways. GO analysis revealed that most DEGs were enriched in immune‐related pathways, such as B‐cell immunity, humoral immune response mediated by circulating immunoglobulin, and complement activation. The above results suggest that a better prognosis of ccRCC patients in the low‐risk group may be caused by the anticancer effects of immune‐related pathways.

### 3.2 Analysis of ERLR‐based score and different clinical characteristics

**FIGURE 2:**
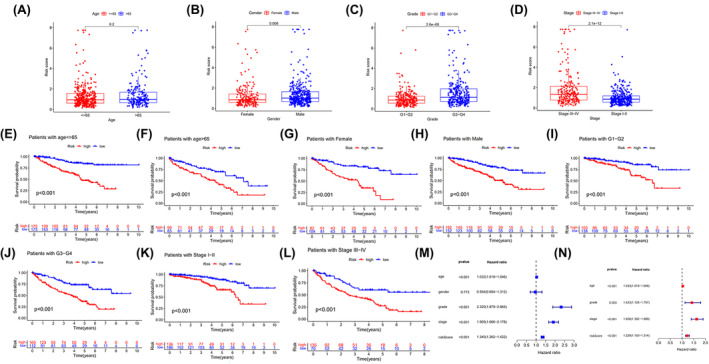
Analysis of ERLR‐based score and different clinical characteristics. (A–D) The correlation between the 4‐ERLR‐based score and the clinicopathological features, including age, sex, grade, and stage. (E–L) Kaplan–Meier survival curves for low‐risk and high‐risk groups; patients were divided into two groups according to different clinicopathological classifications: age, gender, grade, stage. (M, N) Clinical variables and 4‐ERLR‐based score in a univariate or multivariate Cox regression analysis.

For further analysis, we identified several correlations between 4‐ERLR‐based score and clinical features, including gender, age, tumor stage, and grade. Results indicated that ERLR‐based score was higher in male ccRCC patients, those with a tumor histology grade of 3–4 and stage III‐IV (Figure 2B–D), regardless of age (Figure 2A). Based on four clinical variables, we divided all patients into several subgroups to display survival using Kaplan–Meier survival analysis and found that high‐risk groups had worse survival across different subpopulations. We confirmed that the 4‐ERLR‐based score had accurate predictive ability for ccRCC patients with different ages (≤65 or >65), different genders (female or male), different tumor histology grades (1–2 or 3–4), and different stages (I‐II or III‐IV) (Figure 2E–L). These results implied that the ERLR‐based score may be intimately associated with tumor progression and could predict the outcome of multiple subgroups of ccRCC patients.

Then, univariate Cox regression analysis and multivariate Cox regression analysis showed that age, tumor stage grade, and a 4‐ERLRs score were significantly correlated with the prognosis of ccRCC patients (Figure 2M,N). Therefore, 4‐ERLR‐based score is a promising independent prognostic scoring system in ccRCC. Meanwhile, to develop a clinical utility for predicting the survival probability in ccRCC patients, we constructed a nomogram integrating 4‐ERLRs score and clinicopathological features, including age, gender, grade, and stage (Figure S3A,C). The calibration plots displayed a good performance in 1‐, 2‐, and 3‐year overall survival, indicating that the nomogram had good accuracy as an ideal model (Figure S3B,D). The nomogram predicted the probability of 1, 3, and 5 years in ccRCC patients, combined with the ERLR‐based score, providing a quantitative method for predicting the OS in ccRCC patients, and acting as a valuable clue for clinicians to make medical decisions and follow‐up plans.

### 3.3 Correlation of 4‐ERLR‐based score with immune infiltration

**FIGURE 3:**
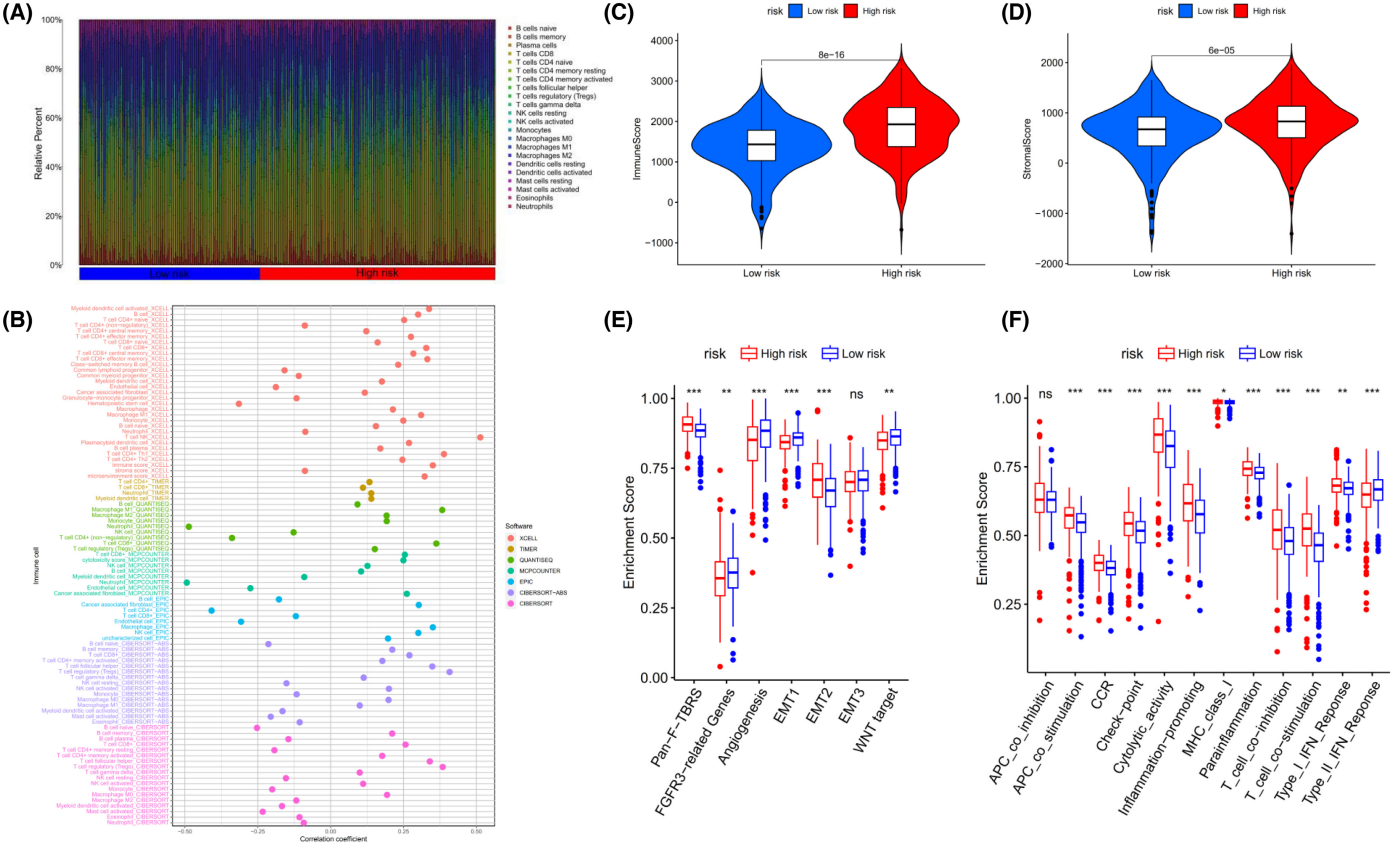
Correlation of 4‐ERLR‐based score with immune infiltration. (A) Proportions of 22 immune cell types in ccRCC patients. (B) Correlations between ERLRs‐based score and immune cell infiltrations of ccRCC by the following software: XCELL, TIMER, QUANTISEQ, MCPCOUNTER, EPIC, CIBERSORT‐ABS, and CIBERSORT. (C) ImmuneScore for the low‐risk and high‐risk groups. (D) StromalScore for the low‐risk and high‐risk groups. (E) Correlation of the score with seven stromal‐related pathways. (F) Correlation of the score with 13 immune‐related functions. *p < 0.05, **p < 0.01, ***p < 0.001 and ns, no significance.

To investigate the roles of the 4‐ERLR‐based score in the immune microenvironment of ccRCC patients, ESTIMATE algorithm was used to further investigate the associations between the 4‐ERLR‐based score and immune infiltration cells. Proportions of various immune cells were shown in Figure 3A, which intuitively reflected immune cells pattern differences in the low‐ and high‐risk groups. T cells and macrophages accounted for the largest components. Correlation between immune infiltration status and riskScore was assessed by seven different types of software (Figure 3B).

Then, we assessed immune infiltration status in the low‐ and high‐risk groups. We estimated the proportions of 22 immune cell types in ccRCC by running the CIBERSORT script and found that CD8 + T cells, T follicular helper cells (Tfh), and regulatory T cells (Tregs) were significantly enriched in the high‐risk group. Monocytes, macrophages M0, macrophages M1, resting dendritic cells, activated dendritic cells, and resting mast cells were mainly enriched in the low‐risk group (Figure S4A). Meanwhile, similar outcomes were found by running the ESTIMATE script (Figure S4B). At the same time, the immune score, tumor microenvironment interstitial score, and estimate score of the high‐risk group were statistically higher, indicating that the high‐risk group may be more sensitive to immunotherapy than low‐risk group (Figure 3C,D) (Figure S4E). Moreover, infiltration levels of all 10 significant immune‐associated gene sets were higher in high‐risk ccRCC patients, including APC‐co‐stimulation, CCR, checkpoint, cytolytic activity, inflammation‐promoting, MHC class I, parainflammation, T‐cell co‐inhibition, T‐cell co‐stimulation, Type I IFN Response, except for Type II IFN Response (Figure 3F). These findings suggest that the immune function of high‐risk ccRCC individuals was more active than that of low‐risk ccRCC individuals. Correlations between stromal‐related pathways and the 4‐ERLR‐based score were also analyzed. Among them, the high‐risk group was correlated with Pan‐F‐TBRS and EMT2, while the low‐risk group was associated with FGFR‐related genes, angiogenesis pathways, EMT1 and WNT targets (Figure 3E). Meanwhile, immune activation genes were upregulated in the high‐risk group (Figure S4C). Differences in TGFβ and EMT pathway‐related gene expression levels of the two groups are shown in Figure S4D, suggesting that the expression levels of PDGFRA, TWIST1, and VIM were increased in the high‐risk group, indicating a poorer prognosis in ccRCC patients.

In conclusion, the high‐risk group had higher levels of immunosuppressive cell, suggesting that the immune function of these cells was possibly impaired. At the same time, the high‐risk group had a potential state of increasing vascularization and a worse prognosis. This result provides potential immunotherapy targets and therapeutic directions.

### 3.4 Association between the 4‐ERLR‐based score and somatic mutation status

**FIGURE 4:**
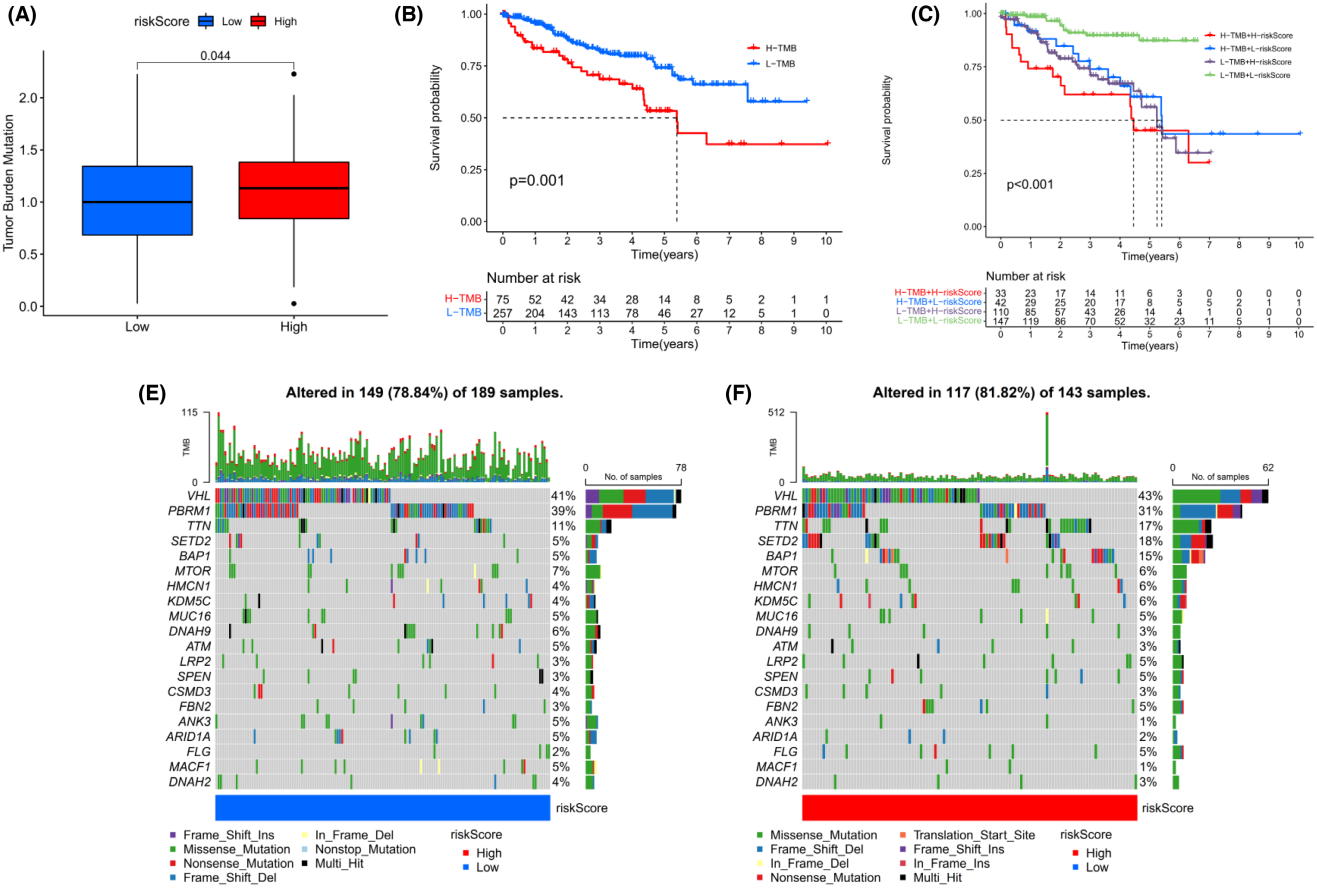
Association between 4‐ERLR‐based score and somatic mutation status. (A) TMB between low‐ and high‐ risk groups. (B) Kaplan–Meier curve showing survival of ccRCC patients in different TMB groups. (C) Kaplan–Meier curve showing survival of patients in different TMB and riskScore groups. (D) Mutation rates and types of top 20 genes in the low‐risk group. (E) Mutation rates and types of top 20 genes in the low‐risk group. (F) Mutation rates and types of top 20 genes in the high‐risk group.

Since the tumor mutation burden (TMB) has been recognized as a predictive biomarker for immunotherapy response in cancer, we further explored the correlation between the TMB and 4ERLRs‐based score. TMB was higher in the high‐risk group, indicating that tumors with more mutations were enriched in the high‐risk group, leading to a worse prognosis (Figure 4A,B). Combined with TMB and riskScore, high‐TMB + low‐RiskScore is slightly better than low‐TMB + high‐RiskScore (Figure 4C), so it can be concluded that riskScore plays a more significant role than TMB, and is more valuable when predicting survival and is related to the amount of tumor mutations.

We then analyzed distributional differences of somatic mutations between the low‐ and high‐risk groups. We found that VHL and PBRM1 had similar mutation frequencies and the two highest mutation frequencies in patients with ccRCC were in SETD2 and BAP1, more than three times higher in the high‐risk group than in the low‐risk group (Figure 4D,E). These would be valuable to reveal underlying mechanisms by which mutations in BAP1 and SETD2 exert diverse effects on cancer progression in ccRCC. The 4‐ERLR‐based score might have implications for predicting immunotherapeutic outcomes in ccRCC.

### 3.5 Association between the 4‐ERLR‐based score and immunotherapy sensitivity

**FIGURE 5:**
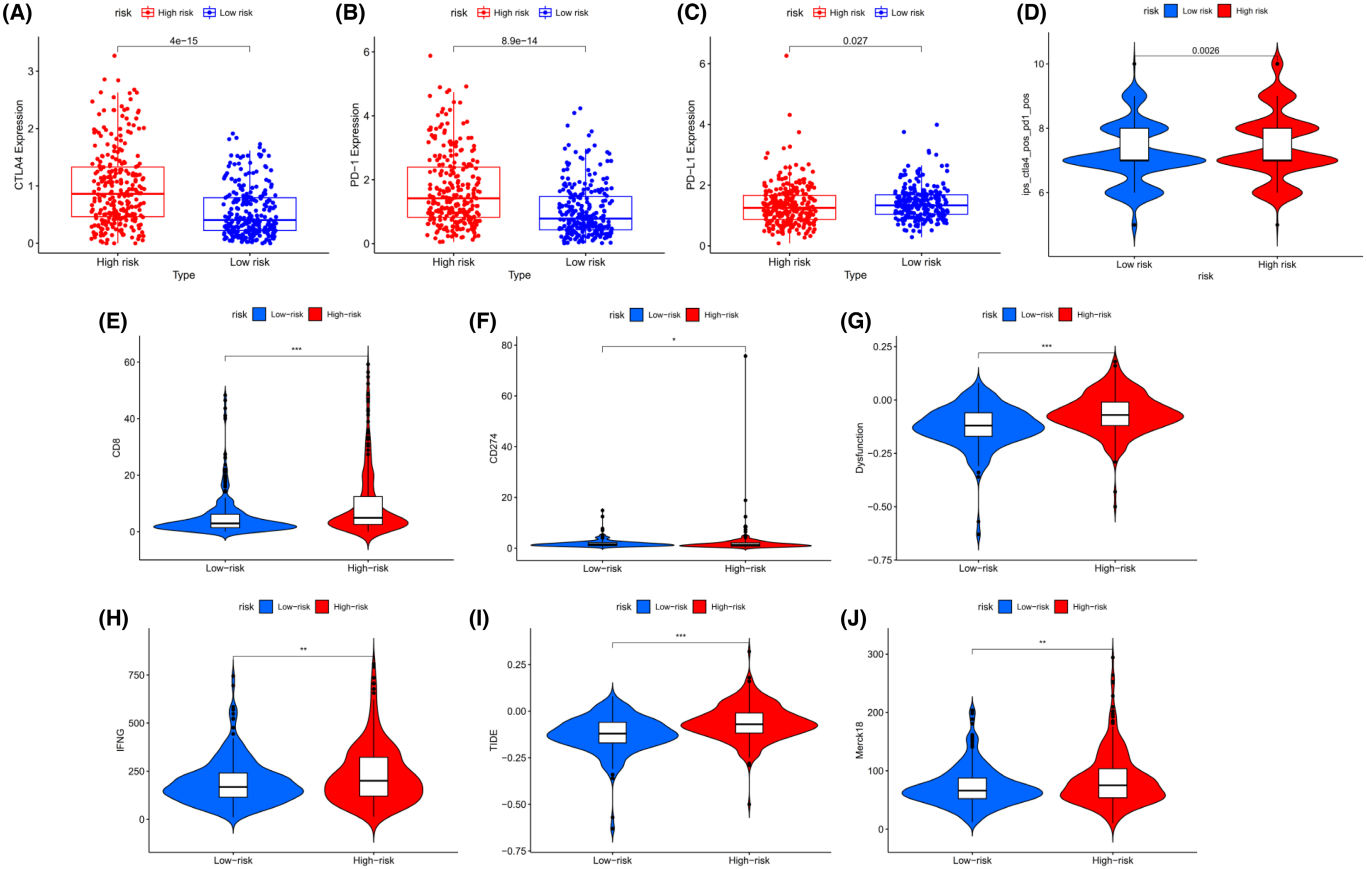
Associations between 4‐ERLR‐based score and immunotherapy sensitivity. (A–C) Distribution of normalized expression levels of the CTLA4, PD‐1, and PD‐L1 across low‐ and high‐risk groups. (D–J) Prediction of immunotherapy efficacy for low‐ and high‐risk groups. *p < 0.05, **p < 0.01, ***p < 0.001.

Immune checkpoint inhibitor (ICI), as a kind of tumor immunotherapy, is a promising strategy for ccRCC. Immune checkpoint molecules include PD‐1, PD‐L1, cytotoxic T lymphocyte‐associated protein 4 (CTLA‐4), T‐cell immunoglobulin mucin‐3 (TIM‐3), and others. The expression levels of PD‐1 and CTLA4 were upregulated in the high‐risk group, indicating that patients in the high‐risk group were promising candidates for anti‐PD1 and anti‐CTLA4 immunotherapy (Figure 5A–C). At the same time, ips_ctla4_pos_pd1_pos, CD8 score, and CD274 score were higher in the high‐risk group than in the low‐risk group, suggesting that patients in the high‐risk group were more likely to benefit from immunotherapy (Figure 5D–F). Moreover, dysfunction score, TIDE, and Merck 18 are higher in the high‐risk group, indicating that the potential immune escape is stronger and more cytotoxic T lymphocyte infiltration in the high‐risk group (Figure 5D–J). Notably, IFNG scores also are higher in the high‐risk group (Figure 5K). In conclusion, the poor prognosis of high‐risk ccRCC patients may be due to tumor immune evasion which could contribute to tumor invasion and metastasis.

### 3.6 Construction and analysis of a lncRNA‐miRNA‐mRNA ceRNA network

**FIGURE 6:**
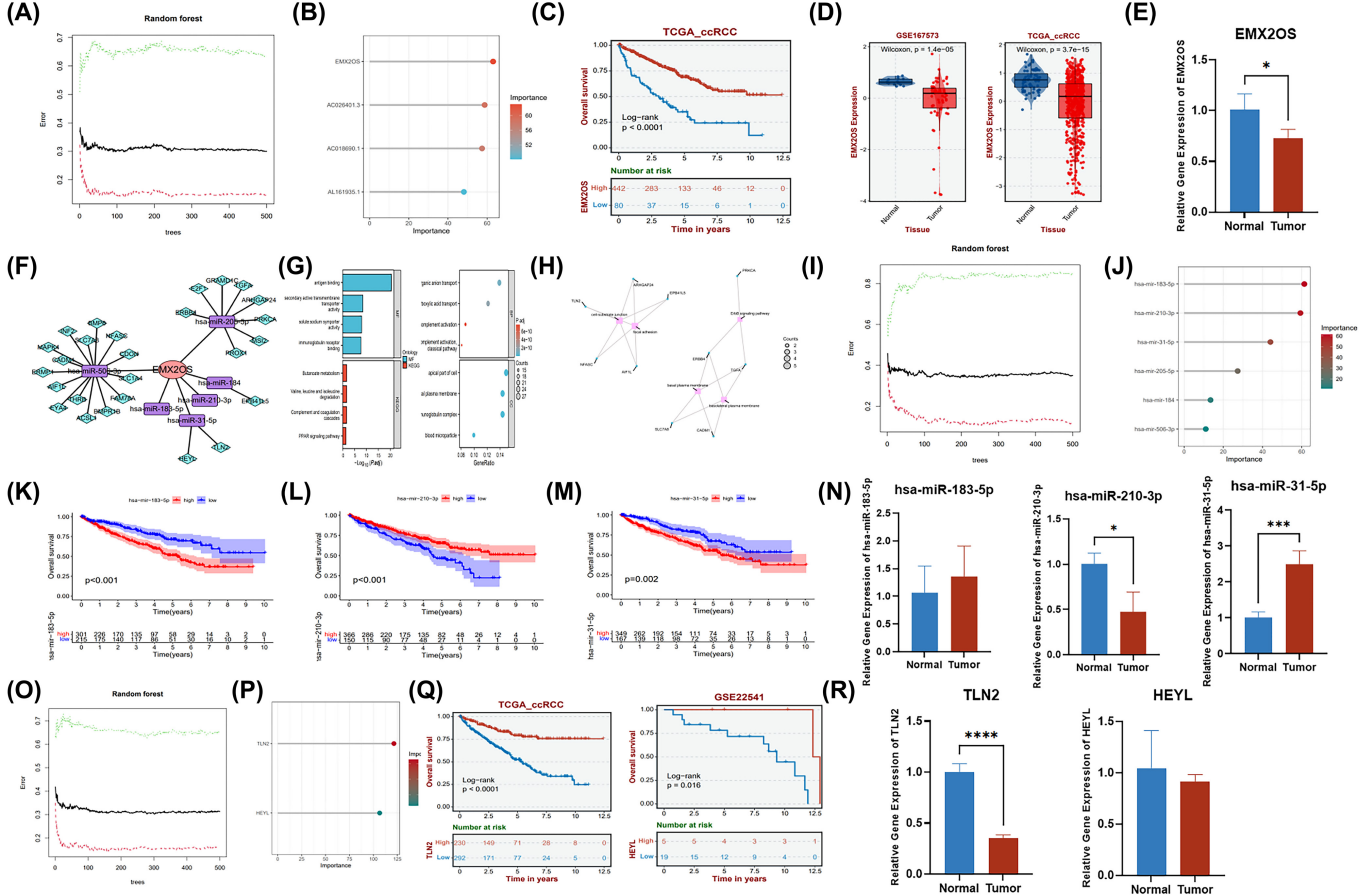
Construction and analysis of a lncRNA‐miRNA‐mRNA ceRNA network. (A, B) The importance of 4ERLRs by machine‐learning analysis. (C) Kaplan–Meier curve showing the overall survival of ccRCC patients in low EMX2OS expression and high EMX2OS expression subgroups. (D) The expression levels of EMX2OS in validation set GSE167573 and TCGA_ccRCC. (E) The expression level of EMX2OS detected by RT‐qPCR in clinical sample. (F) EMX2OS‐associated ceRNA network in ccRCC. (G, H) The relevant GO and KEGG enrichments of the co‐expressed DEGs involved in the ceRNA network. (I, J) The importance of downstream miRNAs of EMX2OS by machine‐learning analysis. (K–M) Kaplan–Meier curve showing the overall survival of ccRCC patients with different hsa‐miR‐183‐5p, hsa‐miR‐210‐3p, and hsa‐miR‐31‐5p expression levels. (N) The expression levels of hsa‐miR‐183‐5p, hsa‐miR‐210‐3p, and hsa‐miR‐31‐5p detected by RT‐qPCR in clinical samples. (O, P) The importance of functional downstream mRNAs of hsa‐miR‐31‐5p by machine‐learning analysis. (Q) Kaplan–Meier curve to show the overall survival of ccRCC patients in different TLN2 and HEYL expression levels. (R) The expression levels of TLN2 and HEYL detected by RT‐qPCR in clinical samples. *n* = 3 in each clinical sample group; data were shown as the Mean ± SD; *p < 0.05, **p < 0.01, ***p < 0.001, ****p < 0.0001; statistical analysis was performed by Student *t*‐test analysis.

The competitive endogenous RNA (ceRNA) is a well‐known regulatory mechanism of lncRNA. Among the 4ERLRs, we found EXM2OS was the first rank of importance‐through mechanical learning (Figure 6A,B). Kaplan–Meier analysis showed that ccRCC patients with high EMX2OS expression had better survival (Figure 6C). In addition, we found that EMX2OS was significantly downregulated in the ccRCC validation set GSE167573 and TCGA_ccRCC (Figure 6D), which was also validated in clinical samples (Figure 6E). Then, we constructed a ceRNA network composed of EMX2OS, differentially expressed miRNAs (DEmiRNAs), and differentially expressed mRNAs (DEmiRNAs) based on multiple databases. The network diagram was visualized by Cytoscape, in which EMX2OS, 6 DEmiRNAs, and 26 DEmRNAs were shown as nodes and 32 interactions were shown as edges (Figure 6F). Meanwhile, analysis of the KEGG signaling pathway and GO analysis revealed that the DEGs were mainly enriched in peroxisome proliferator‐activated receptor (PPAR) signaling pathway and complement activation classical pathway, with a close relationship to regulated amino acid metabolism, regulation of transmembrane transport, and regulation of receptor‐mediated immunity. Furthermore, five significantly correlated GO and KEGG items were determined and visualized according to genes (Figure 6G,H). Then, we found that hsa‐miR‐183‐5p, hsa‐miR‐210‐3p, and hsa‐miR‐31‐5p are more important in DEmiRNAs using mechanical learning (Figure 6I,J). Kaplan–Meier analysis showed that ccRCC patients with low expression of hsa‐miR‐183‐5p or hsa‐miR‐31‐5p had better survival, while the high expression group of hsa‐miR‐210‐3p had better survival (Figure 6K–M). The expression of hsa‐miR‐31‐5p was significantly increased in clinical samples (Figure 6N). Next, we found that TLN2 played a key role as the downstream mRNA of hsa‐miR‐31‐5p by machine learning (Figure 6O,P). Kaplan–Meier analysis also showed that ccRCC patients with high TLN2 or HEYL expression had better survival. We found that TLN2 was significantly downregulated in the ccRCC validation set GSE167573 and TCGA_ccRCC (Figure S6A–C), which was also validated in clinical samples (Figure 6Q,R). Therefore, our study found that the downregulation of EMX2OS and TLN2 may be related to the poor prognosis of ccRCC. The EMX2OS/hsa‐miR‐31‐5P/TLN2 axis might yield functional impact on tumor microenvironment in ccRCC and may represent a novel therapeutic target for ccRCC.

### 3.7 Drug sensitivity and immunotherapy analysis of the ceRNA network

**FIGURE 7:**
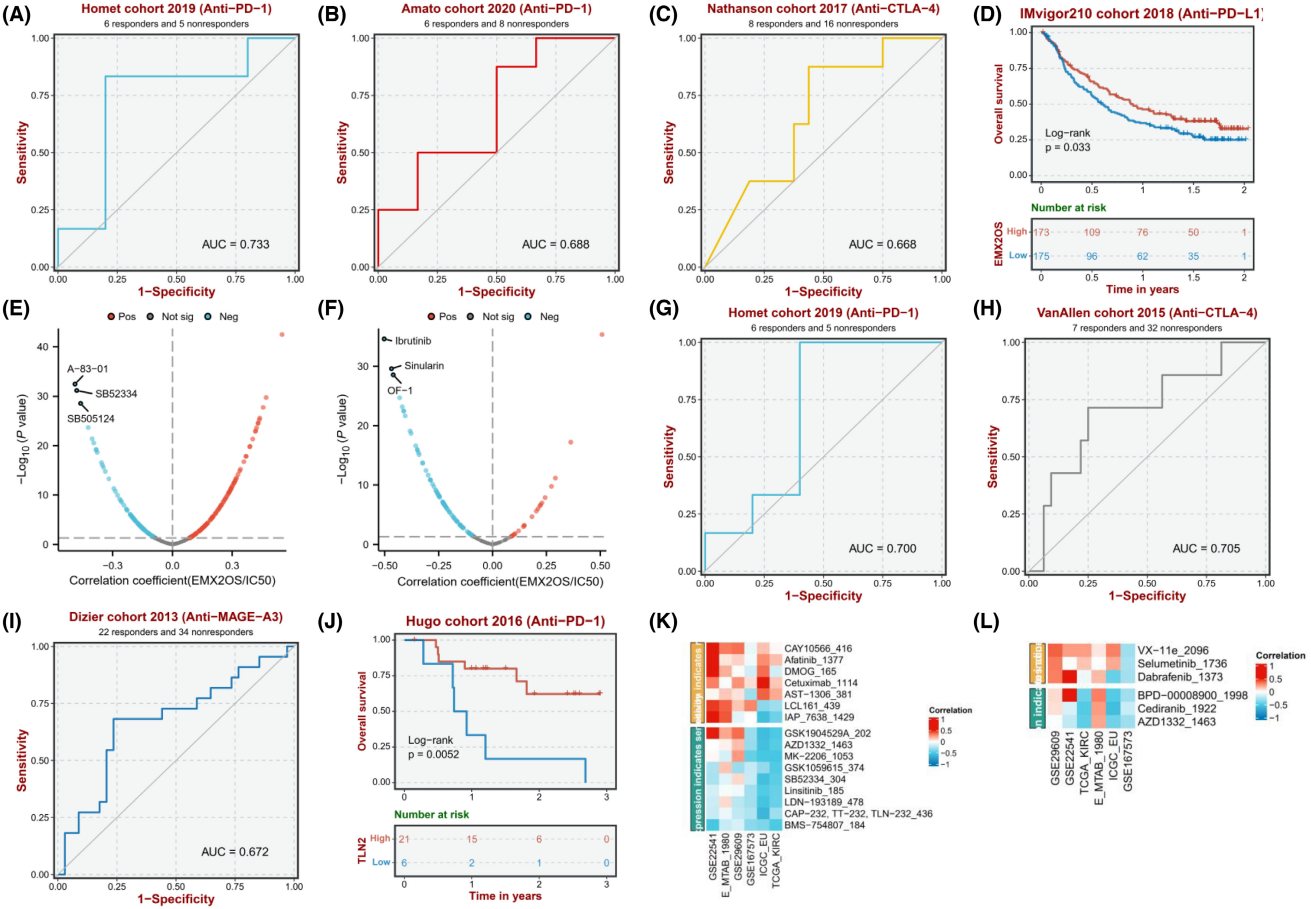
Drug sensitivity and immunotherapy analysis of the ceRNA network. (A–C) ROC curves of anti‐PD1 and anti‐CTLA4 based on EMX2OS expression. (D) Kaplan–Meier curve showing the overall survival of anti‐PD1 based on EMX2OS expression. (E, F) Drug sensitivity analysis of low EMX2OS expression and high EMX2OS expression subgroups. (G–I) ROC curves of anti‐PD1, anti‐CTLA4, and anti‐MAGE‐A3 based on TLN2 expression. (J) Kaplan–Meier curve showing the overall survival of anti‐PD1 based on TLN2 expression. (K, L) Drug sensitivity analysis of low TLN2 expression and high TLN2 expression subgroups.

For EMX2OS, it is found that the anti‐PD‐1 AUC is 0.733 and 0.688 in the Homet cohort 2019 and Amato cohort 2020, respectively. The Anti‐CTLA‐4 AUC is 0.668 in the Nathanson cohort 2017 (Figure 7A–C). For TLN2, it is found that the anti‐PD‐1 AUC is 0.700 and 0.679 in the Homet cohort 2019 and Ascierto cohort 2016, respectively. The anti‐CTLA‐4 AUC is 0.705 in the VanAllen cohort 2015. In addition, the anti‐MAGE‐A3 AUC is 0.672 in the Dizier cohort 2013 (Figure 7G,I). Therefore, we found that the response to anti‐PD‐1 and anti‐CTLA4 treatments was closely correlated with the expression levels of EMX2OS and TLN2 in ccRCC patients (Figure 7D,J). Meanwhile, lower expression of EMX2OS or TLN2 was significantly associated with longer disease‐free survival, shorter disease‐specific survival, and shorter progression‐free survival (Figure S5A–C, G–I). Then, according to the results of drug susceptibility test (Figure 7E,F,K,L), we found that sensitivity to 6 drugs (A‐83‐01, SB52334, SB505124, Ibrutinib, Sinularin, OF‐1) for ccRCC was negatively associated with expression of EMX2OS. Moreover, we found sensitivity to six drugs (AZD1332, MK‐2206, SB52334, Linsitinib, LDN‐193189, BMS‐754807, and Cediranib) for ccRCC was negatively associated with expression of TLN2. Altogether, it appears that these drugs would have higher sensitivity in ccRCC and improve prognostic status and survival in ccRCC patients.

## 4 DISCUSSION

Clear cell renal cell carcinoma is one of the most common malignancies of the urinary tract, accounting for approximately 2.2% of all cancer incidences, and its occurrence continues to increase.^13^ Surgery is the preferred treatment for ccRCC; however, due to the high aggressiveness and drug resistance of ccRCC, 20%–40% of patients with ccRCC experience recurrence and metastasis after surgery.^39^ More studies have found that precision medicine is the key to improving ccRCC outcomes; however, because of the lack of reliable biomarkers, precision medicine is severely limited in the treatment of ccRCC.^40^, ^41^, ^42^ Exosomes have the advantages of easy availability, non‐invasive examination, and tumor specificity.^24^ Meanwhile, due to their high mobility and lipid bilayer structure, they can easily passthrough biological membranes and protect rich bioactive substances present inside the membranes from degradation.^43^ Tumor‐derived exosomes can carry a large number of substances, including proteins, nucleic acids, and lipids, among them, exosome lncRNAs not only play a role in ccRCC diagnosis and prognosis, but also can be used as therapeutic targets, playing a crucial role in ccRCC progression and the spread of drug resistance.^44^

Therefore, through various mechanical learning methods, we screened out four key exosome‐related lncRNAs (EMX2OS, AC026401.3, AC018690.1, and AL161935.1) and constructed the four exosome‐related lncRNAs (4‐ERLR)‐based score for ccRCC. Among these four lncRNAs, EMX2OS and AC026401.3 have been closely correlated with ccRCC progression in previous studies. Jiang et al. found EMX2OS may regulate energy metabolism by enriching the FOXO and PPAR signaling pathways to influence the prognosis of ccRCC.^45^ Deng et al. found the expression of AC026401.3 tended to be higher in ccRCC tissues, and AC026401.3 may be critically associated with the differentiation and maturation of tumor‐infiltrating lymphocytes.^46^ As for AC018690.1 and AL161935.1, the relative information could not be available because of the little research on these two novel lncRNAs. Meanwhile, our results showed that the 4‐ERLR‐based score might be an independent prognostic factor for ccRCC patients and patients with a high score in the 4ERLRs‐based score had a worse survival rate. The score has outperformed traditional clinical characteristics in terms of ccRCC survival prediction. We also identified that the nomogram including the 4‐ERLR‐based score showed a high performance in 1, 3, and 5 years, which may help in the analysis of the prognosis of ccRCC patients and guide the choice of treatment. Moreover, the 4ERLRs‐based score was intimately associated with tumor mutation burden (TMB), suggesting that they could potentially help clinicians design effective individual therapy for ccRCC patients. BAP1 and SETD2 had a high mutation rate in the high‐risk group, mainly encoding proteins involved in chromatin modification and remodeling, and co‐deletions with VHL were common and important co‐drivers of tumorigenesis. These genes are all located near VHL on chromosome 3p and are often altered following cytogenetic rearrangements that lead to 3p loss and precede the establishment of ccRCC.^47^, ^48^, ^49^ These can suggest potential therapeutic strategies for patients with ccRCC characterized by deficiencies in these proteins.

In order to better understand the ERLRs potential mechanism of patients with ccRCC, we found that EMX2OS played the most important value among the 4ERLRs through mechanical learning. On this basis, we built the relevant ceRNA network and found that EMX2OS might have an important function in the ccRCC tumor microenvironment through the EMX2OS/hsa‐miR‐31‐5P/TLN2 axis. Reportedly, hsa‐miR‐31a‐5p plays a role in the generation of oxidative stress, and it is upregulated in the urinary exosomes of patients with diabetic nephropathy.^50^ Another study showed that miR‐31‐5p was found to be declined in patients with diabetic nephropathy and had a suppressive effect on the apoptosis, inflammation, and oxidative stress in podocytes under high glucose condition.^51^ Talin is a macromolecular cytoskeleton protein located on the extracellular matrix (ECM) that can bind to a variety of adhesion molecules; studies have shown that two Talin genes, TLN1 and TLN2, in vertebrates, encode Talin 1 and Talin 2 proteins, respectively.^52^ TLN2 has 74% homology with Talin 1. Talin1 can regulate FA dynamics, cell migration, and cell invasion.^53^ Cai et al. found that low levels of TLN2 notably correlated with shorter survival rates and tumor aggressiveness in patients with ccRCC, and TLN2 strongly correlated with ccRCC proliferation, migration, and invasion via regulation of the Wnt/β‐catenin pathway.^54^ However, the regulatory mechanism of their interaction in ccRCC has not been studied. In our study, we found that the high expressions of EMX2OS and TLN2 were tightly associated with improved OS, while the high expression of hsa‐miR‐31‐5p was highly associated with poor OS. Recent studies have pointed out that ERLRs may influence ccRCC development, metabolic, and immune pathways by mediating a ceRNA regulatory network.^55^, ^56^ Therefore, we speculate that EMX2OS may regulate TLN2 by interfering with the function of hsa‐miR‐31‐5p. The EMX2OS‐hsa‐miR‐31‐5p‐TLN2 axis may be the potential therapeutic targets for ccRCC.

Subsequently, we endeavored to estimate the association between the expression level of EXM2OS or TLN2 and drug sensitivity in ccRCC patients. We identified potential drug targets for high‐risk ccRCC patients from the GDSC database, mainly including transforming growth factor‐β (TGF‐β) receptor inhibitors (A‐83‐01, SB52334, SB505124), Brutinib tyrosine kinase (BTK) inhibitors (Ibrutinib), apoptosis related (Sinularin), insulin‐like growth factor‐1 (IGF1) receptor inhibitor (Linsitinib, BMS‐754807), and tyrosine kinase inhibitor (Cediranib). Among them, Ibrutinib is a novel angiogenesis inhibitor via upregulating BMP4 expression leading to vascular endothelial dysfunction.^57^ Meanwhile, Lara et al. found that ibrutinib combined with an immune checkpoint PD1 inhibitor (nivolumab) enhanced anti‐tumor activity in patients with metastatic renal cell carcinoma.^58^ However, the data on the therapeutic efficacy of Ibrutinib in ccRCC are still lacking. Sinularin markedly suppresses cell growth and induces apoptosis, as well as activate MAPKs and repress PI3K/AKT pathways, which are dependent on ROS generation, thereby exerting anti‐tumor effects against human renal cancer cells.^59^ Linsitinib impaired the cell migration and invasion capability of RCC cells by efficiently reversing oncogenic phenotype of RCC cells.^60^ A randomized, double‐blind phase II study for evaluating cediranib in patients with relapsed metastatic clear cell renal cancer (COSAK) illustrated the potential of cediranib for the treatment of ccRCC.^61^ Given that high‐risk ccRCC patients may be more sensitive to the abovementioned drugs and some drugs have achieved certain effects in clinical trials of some solid tumors, the abovementioned drugs may provide new potential therapeutic strategies for the high‐risk subgroup of ccRCC, while providing a possible reference for clinicians to choose.

Finally, immunotherapy is currently viewed as a breakthrough in ccRCC treatment that has demonstrated promising results in improving the prognosis of ccRCC patients across the globe.^62^, ^63^ The tumor microenvironment, which contains extracellular matrix, fibroblasts, endothelial cells, and multiple immune cells, plays a critical role in tumor progression, immune escape, and responses to therapies, especially immunotherapies.^64^ In our study, we found that the high‐risk group mainly enriched CD8^+^T cells, T follicular helper cells (Tfh), and regulatory T cells (Tregs). Unlike the vast majority of cancers, CD8^+^T‐cell infiltration level was reported as an adverse prognostic factor in ccRCC.^65^ Dai et al. found CXCL13^+^CD8^+^T cells were a highly exhausted subtype of intratumoral CD8^+^T cells with impaired immune function and exhausted markers, and high infiltration of CXCL13^+^CD8^+^T cells in ccRCC could also demonstrate the immunoevasive contexture with an impaired CD8+T‐cell immunity, more pro‐tumor cells and fewer anti‐tumor factors.^66^ Treg cell for cancer immunotherapy is a double‐edged sword. Although the attenuation of the Treg‐mediated suppressive activity increases the anti‐tumor immune response, Treg cell dysfunction is often associated with autoimmune and inflammatory diseases.^67^ Therefore, ccRCC patients with high‐risk group were prone to immune escape, which affects the progression and prognosis of ccRCC. Moreover, we also found that the high‐risk group had higher immune score and tumor microenvironment interstitial score, suggesting that the high‐risk group may be more sensitive to immunotherapy. We next investigated the connection between the 4‐ERLR‐based score and different immune checkpoints. Surprisingly, the patients in the high‐risk group showed a significant rise in the expression of PD1 and CTLA4, implying that the anti‐PD1 immunotherapy and anti‐CTLA4 immunotherapy may be beneficial for these individuals. It is therefore possible that the 4ERLRs‐based score may be used to identify ccRCC patients who are more likely to respond to anti‐tumor immunotherapies. Moreover, we also found that the expression levels of EMX2OS and TLN2 were significantly reduced in ccRCC patients, and the expression levels of EMX2OS and TLN2 were significantly associated with anti‐PD1 immunotherapy and anti‐CTLA4 immunotherapy. These results further confirmed the potential of the 4‐ERLR‐based score in predicting the suitability of immune checkpoint blockade in ccRCC patients, and the high sensitivity of anti‐PD1 immunotherapy and anti‐CTLA4 immunotherapy in ccRCC patients with high‐risk group.

However, tissue biopsy sampling for kidney disease, although highly accurate, is an invasive surgery.^68^ In recent years, researchers have found that the combination of artificial intelligence, new imaging technologies, and biomarkers can better assist clinicians in predicting clinical outcomes and metastasis risks of ccRCC before surgery.^69^, ^70^ Therefore, we envision combining the key lncRNA EMX2OS screened in this article with radiogenomics to achieve more accurate non‐invasive diagnosis and aid in clinical applications. But these all require further experiments to verify and require further research.

In summary, the strength of this study lies in the identification and construction of a new 4‐ERLR‐based score that can feasibly and efficiently evaluate survival and immunotherapy response in ccRCC, and these ERLRs have the potential to be biomarkers and relevant immunotherapy targets for ccRCC patients. Second, we also explored the key lncRNA (EMX2OS)‐associated ceRNA network and found that the EMX2OS‐hsa‐miR‐31‐5P‐TLN2 axis could be considered as a precise potential target for future ccRCC therapy. Finally, this study found that high‐risk ccRCC patients are highly sensitive to anti‐PD1 immunotherapy and anti‐CTLA4 immunotherapy, and at the same time provide a possible new drug regimen for ccRCC patients and offer a reference for clinical combination drug use.

## 5 CONCLUSION

We performed multiple machine‐learning methods and established a clinically instructional ERLR‐based score in ccRCC patients that predicts the prognosis of ccRCC patients. Moreover, we selected the key lncRNA EMX2OS among 4 ccRCC‐related ERLRs and found that EMX2OS might play an important role in the tumor microenvironment through a potential EMX2OS/hsa‐miR‐31‐5p/TLN2 regulatory axis, influencing the survival of patients with ccRCC and potentially guide clinical medication and immunotherapy, thereby providing a clue for precise treatment of ccRCC patients.

## AUTHOR CONTRIBUTIONS

**Zhan Yang:** Conceptualization (lead); data curation (equal); formal analysis (equal); investigation (lead); methodology (lead); resources (lead); validation (equal); writing – original draft (lead). **Xiaoting Zhang:** Conceptualization (lead); data curation (lead); investigation (lead); resources (equal); software (equal); supervision (equal); validation (lead); visualization (lead); writing – original draft (lead). **Ning Zhan:** Conceptualization (lead); project administration (lead); resources (lead); supervision (equal); visualization (equal); writing – review and editing (equal). **Lining Lin:** Data curation (equal); formal analysis (equal); investigation (equal); methodology (equal); resources (equal); software (equal); validation (equal); writing – original draft (supporting). **Jingyu Zhang:** Investigation (lead); resources (lead); supervision (lead); writing – review and editing (lead). **Lianjie Peng:** Conceptualization (equal); formal analysis (equal); methodology (lead); validation (lead); visualization (lead). **Tao Qiu:** Data curation (equal); investigation (equal); resources (equal). **Yaxian Luo:** Conceptualization (equal); methodology (equal); resources (equal). **Chundi Liu:** Data curation (equal); formal analysis (equal); investigation (equal). **Chaoran Pan:** Data curation (equal). **Junhao Hu:** Software (equal). **Yifan Ye:** Conceptualization (equal). **Zilong Jiang:** Investigation (equal). **Xinyu Liu:** Conceptualization (equal). **Mouyuan Sun:** Conceptualization (equal); data curation (equal); formal analysis (equal); funding acquisition (equal); supervision (equal); visualization (equal); writing – review and editing (equal). **Yan Zhang:** Conceptualization (equal); data curation (equal); formal analysis (lead); funding acquisition (lead); methodology (lead); software (lead); supervision (lead); visualization (lead); writing – review and editing (lead).

## FUNDING INFORMATION

This work was financially supported by the following programs: China Postdoctoral Science Foundation (2023 M743009), Zhejiang University of Stomatology Postdoctoral Scientific Research Foundation (2023PDF013), and Zhejiang Provincial Natural Science Foundation of China (LY21H050006).

## CONFLICT OF INTEREST STATEMENT

All authors declare no conflicts of interest in this study.

## ETHICS STATEMENT

These studies were performed in compliance with an approved protocol and the institutional guidelines of the Human Research Ethics Committee of the First Affiliated Hospital of Wenzhou Medical University (Ethics number KY2021‐160) (approval date: 2022–01‐20). All human samples used in this study have obtained written informed consent and have been approved by the Ethics Committee.

## CONSENT FOR PUBLICATION

All authors have reviewed the final version of the manuscript and approved its submission.

## Supporting information


Figure S1:
Figure S2:
Figure S3:
Figure S4:
Figure S5:
Figure S6:

## Data Availability

The datasets generated and/or analyzed during the current study are available in the TCGA database (https://portal.gdc.cancer.gov/) (TCGA‐BLCA) and GEO database (https://www.ncbi.nlm.nih.gov/) (GSE135337). And the data that support the findings of this study are available from the corresponding author upon reasonable request.
