# Supplementary material for: Exosome‐related lncRNA score: A value‐based individual treatment strategy for predicting the response to immunotherapy in clear cell renal cell carcinoma
Source: Cancer Med. 2024 May 29;13(11):e7308. doi: 10.1002/cam4.7308 (PMC11135019; doi:10.1002/cam4.7308)
Supplement: Supplementary file 1 — Figure S1: Figure S2: Figure S3: Figure S4: Figure S5: Figure S6: [file CAM4-13-e7308-s001.docx]

**Supplementary Information of**

**Exosome-related lncRNA score: a value-based individual treatment strategy for predicting the response to immunotherapy in clear cell renal cell carcinoma**

Zhan Yang^#,1^, Xiaoting Zhang^#,2^, Ning Zhan^#,2^, Jingyu Zhang^2^, Lianjie Peng^2^, Lining Lin^2^, Tao Qiu^2^, Yaxian Luo^2^, Chundi Liu^2^, Chaoran Pan^1^, Junhao Hu^1^, Yifan Ye^1^, Zilong Jiang^1^, Xinyu Liu^1^, Mouyuan Sun*^,2^, Mengfei Yu^*,2^, Huiming Wang^2^, Yan Zhang*^1^

^1^Department of Urology, The First Affiliated Hospital of Wenzhou Medical University, Wenzhou, Zhejiang Province, China 325015

^2^Stomatology Hospital, School of Stomatology, Zhejiang University School of Medicine, Zhejiang Provincial Clinical Research Center for Oral Diseases, Key Laboratory of Oral Biomedical Research of Zhejiang Province, Cancer Center of Zhejiang University, Hangzhou 310000.

^#^ Zhan Yang, Xiaoting Zhang and Ning Zhan are co-first authors.

^*^ Yan Zhang, Mengfei Yu and Mouyuan Sun are co-corresponding authors.


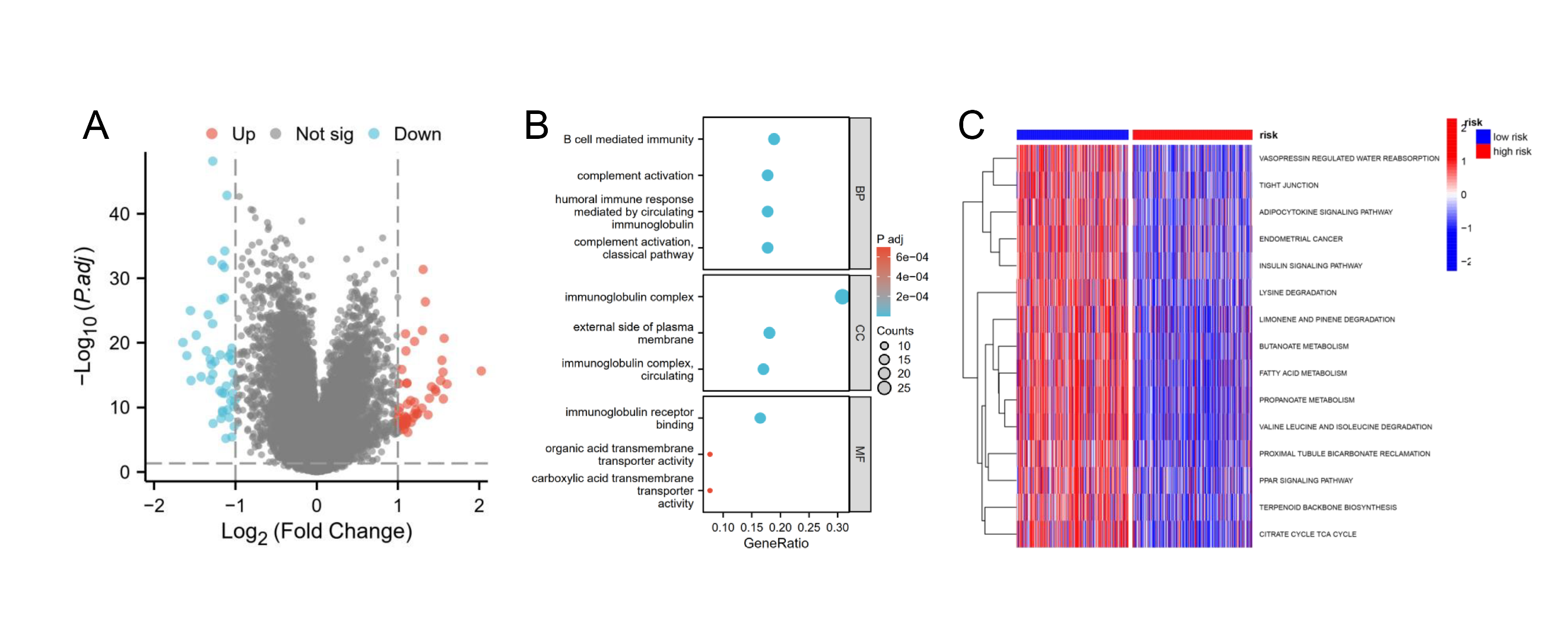


**Figure s2: Comparison of the differentially expressed genes and pathways between the ccRCC high- and low-risk groups**

(A): The differentially expressed genes were screened between the high- and low-risk groups. (B): GO enrichment of differentially expressed genes. (C): Multiple signal pathways were differentially activated between the low- and high-risk groups.


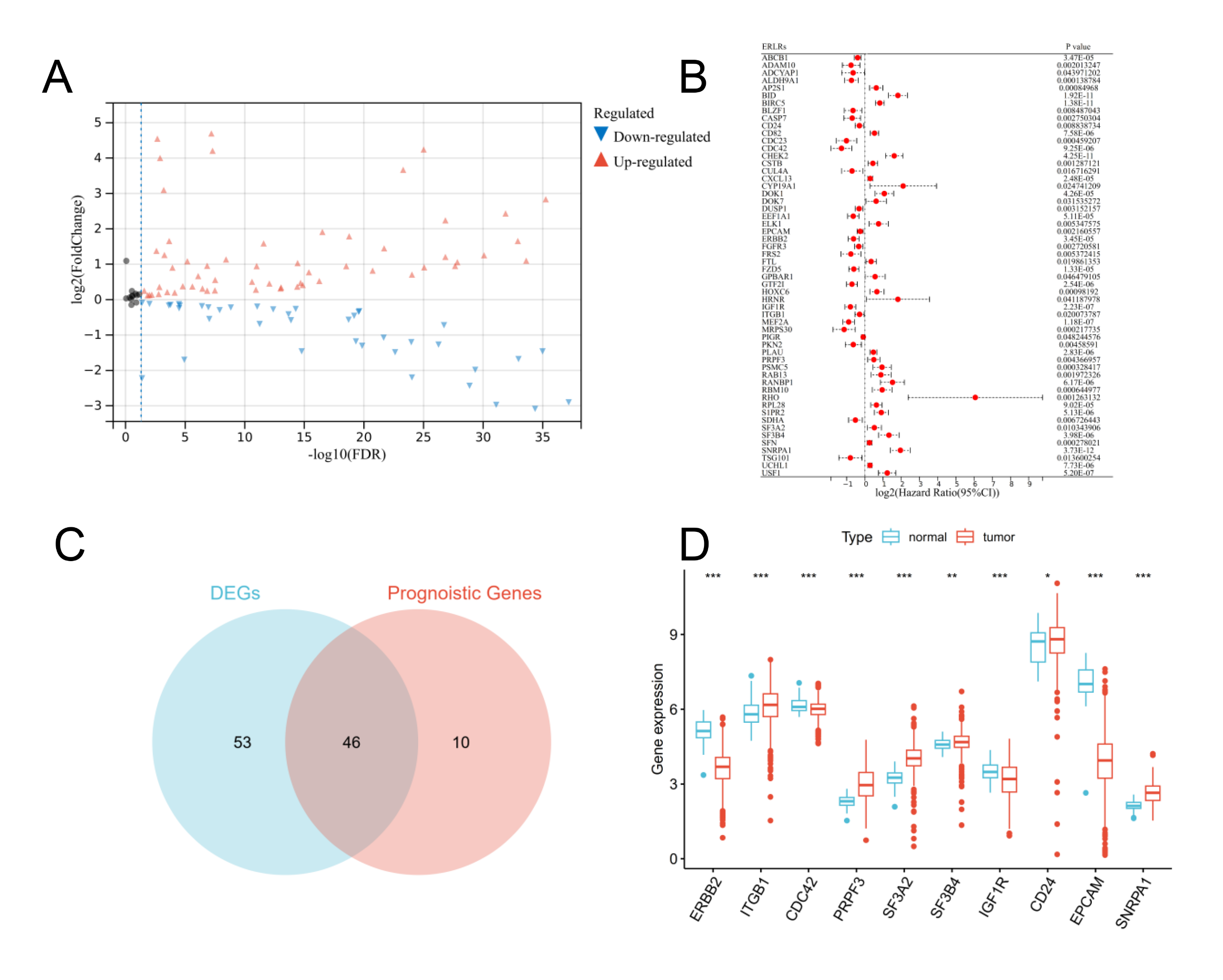


**Figure s1: TOP10 differentially expressed exosomes-related genes**

(A): Differential expression exosomes-related genes in ccRCC. (B): Differentially expressed genes associated with ccRCC prognosis. (C): The Venn diagram revealed the intersection of the exosomes-related DEGs and the prognosis genes. (D): The expression levels of the TOP10 differentially expressed prognostic exosome-related genes. *p<0.05, **p<0.01, ***p<0.001.


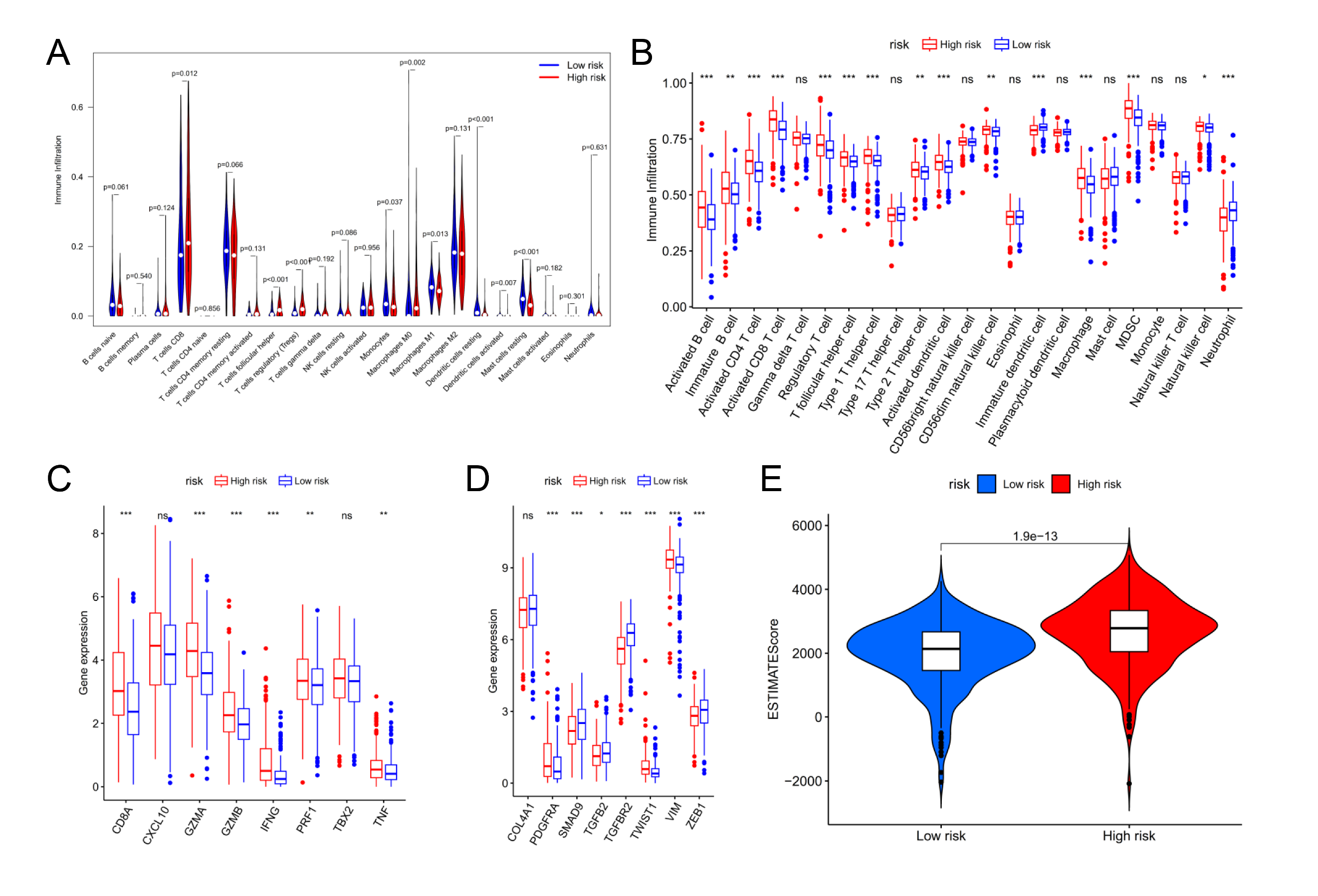


**Figure s4: Analysis of the tumor microenvironment (TME) between the low- and high-risk patients.**

(A): Infiltration levels of 22 immune cells in the low- and high-risk groups using CIBERSORT. (B): Infiltration levels of 23 immune cells in the low- and high-risk groups using ESTIMATE. (C-D): Expression levels of immune-related genes in low- and high-risk groups. €: ESTIMATEScore for the low-risk and high-risk groups. *p<0.05, **p<0.01, ***p<0.001 and ns: no significance.


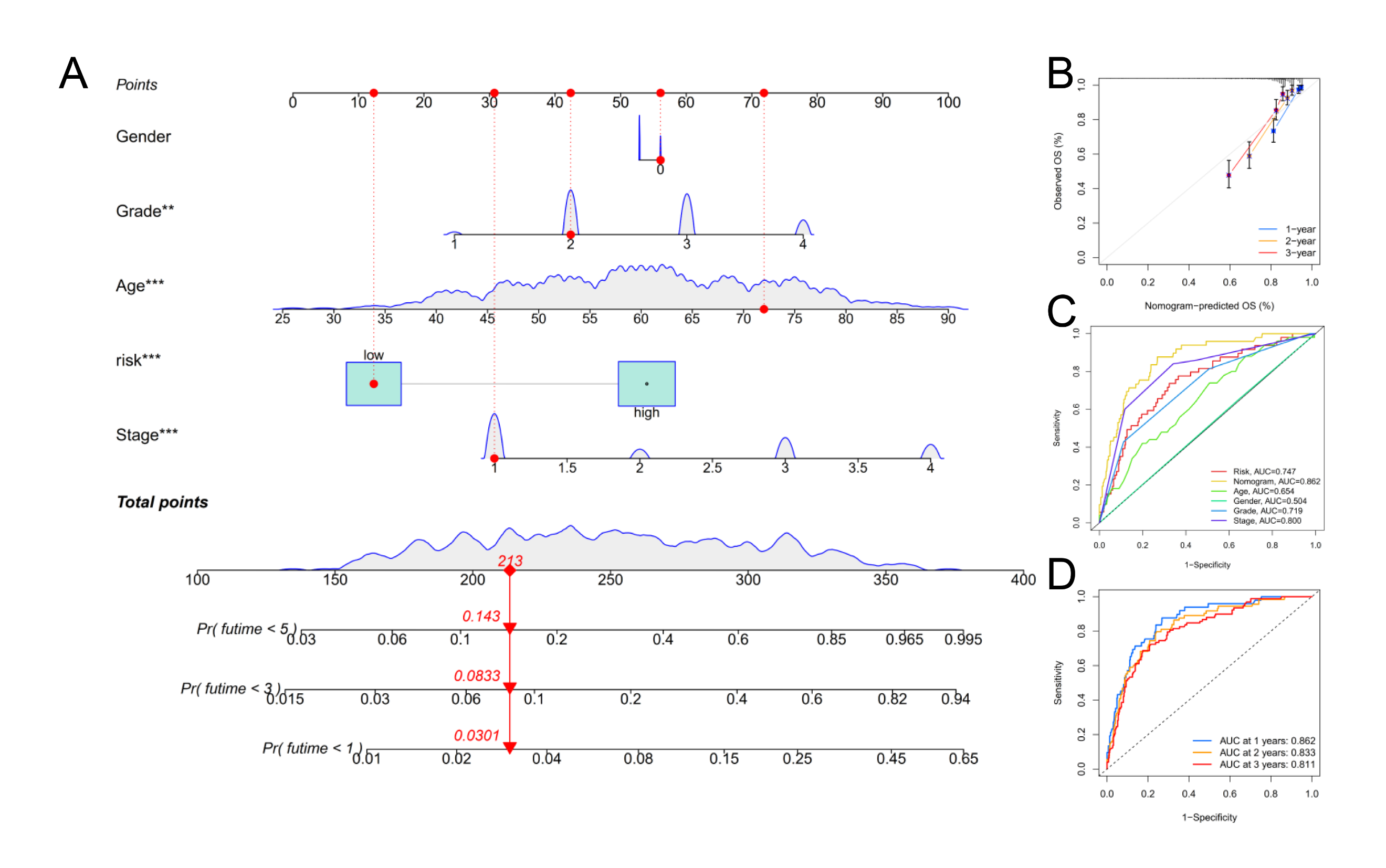


**Figure s3: Clinical nomogram**

(A): A nomogram showing risk and clinicopathological features for predicting 1-, 3- and 5-OS in ccRCC patients. (B): Calibration curves indicating the accuracy of risk model to predict 1-, 2- and 3-OS of ccRCC patients. (C): The prediction of 1-, 2- and 3-OS for ccRCC patients in the entire set. D: Comparison of predictive risk model and clinicopathological features.


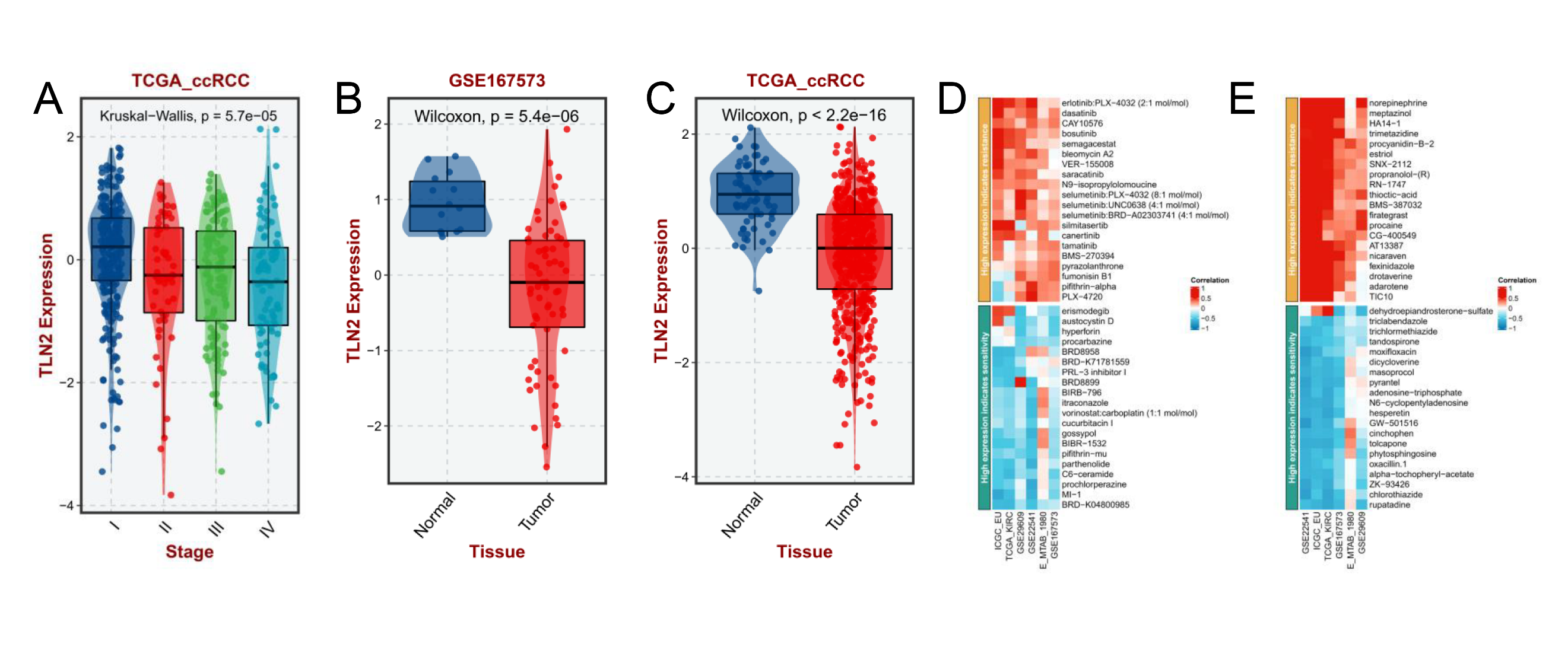


**Figure s6**: Drug sensitivity analysis and expression level validation of TLN2

(A-C): The expression levels of TLN2 in validation set GSE167573 and TCGA_ccRCC. (D-E): Drug sensitivity analysis of low TLN2 expression and high TLN2 expression subgroups.


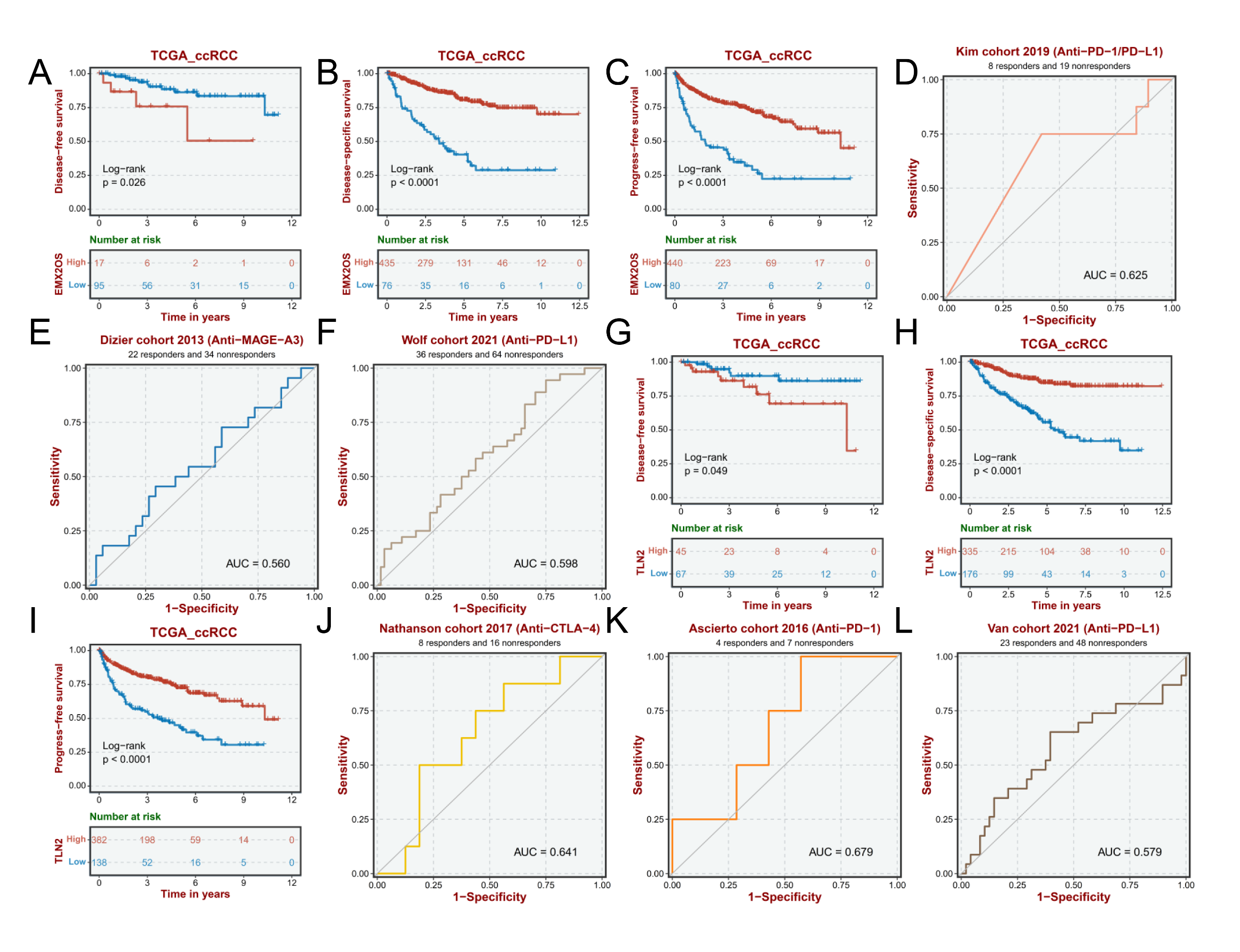


**Figure s5: Immunotherapy analysis based on EMX2OS-associated ceRNA network.**

(A): Kaplan-Meier curve to show DFS of ccRCC patients in different EMX2OS expression levels. (B): Kaplan-Meier curve to show DSS of ccRCC patients in different EMX2OS expression levels. (C): Kaplan-Meier curve to show PFS of ccRCC patients in different EMX2OS expression levels. (D-F): ROC analysis of immune checkpoints based on EMX2OS expression. (G): Kaplan-Meier curve to show DFS of ccRCC patients in different TLN2 expression levels. (H): Kaplan-Meier curve to show DSS of ccRCC patients in different TLN2 expression levels. (I): Kaplan-Meier curve to show PFS of ccRCC patients in different TLN2 expression levels. (J-L): ROC analysis of immune checkpoints based on TLN2 expression. DFS: Disease-free survival; DSS: Disease-specific survival; PSS: progress-free survival.
